# Evidence of whole-body vibration exercises on body composition changes in older individuals: a systematic review and meta-analysis

**DOI:** 10.3389/fphys.2023.1202613

**Published:** 2023-11-02

**Authors:** Aline Reis-Silva, Ana Carolina Coelho-Oliveira, Márcia Cristina Moura-Fernandes, Monteiro-Oliveira Bruno Bessa, Daniel Batouli-Santos, Mario Bernardo-Filho, Danúbia da Cunha de Sá Caputo

**Affiliations:** ^1^ Programa de Pós-Graduação em Ciências Médicas, Faculdade de Ciências Médicas, Universidade do Estado do Rio de Janeiro, Rio de Janeiro, RJ, Brazil; ^2^ Laboratório de Vibrações Mecânicas e Práticas Integrativas, Instituto de Biologia Roberto Alcântara Gomes e Policlínica Universitária Piquet Carneiro, Universidade do Estado do Rio de Janeiro, Rio de Janeiro, RJ, Brazil; ^3^ Programa de Pós-Graduação em Fisiopatologia Clínica e Experimental, Faculdade de Ciências Médicas, Universidade do Estado do Rio de Janeiro, Rio de Janeiro, RJ, Brazil

**Keywords:** systemic vibratory therapy, mechanical vibration, physical exercise, muscle mass, fat mass, health, aging body

## Abstract

**Introduction:** The aging process is associated with changes in body composition, including fat gain and skeletal muscle loss from middle age onward. Moreover, increased risk of functional decline and the development of chronic diseases are also related to aging.

**Objective:** This systematic review and meta-analysis aimed to evaluate the effects of whole-body vibration exercise (WBVE), as a physical exercise, on body composition in people over 60 years of age.

**Methods:** Searches were performed on PubMed, Scopus, Web of Science, and Embase. Only randomized clinical trials evaluating the effects of WBVE on body composition in older individuals were considered. The methodological quality of the studies involved was assessed using the Physiotherapy Evidence Database (PEDro) scale, recommendations from the Cochrane Collaboration were used to assess risk of bias, and quality of evidence was assessed using the Grading of Recommendation, Assessment, Development, and Evaluation (GRADE) methodology. RevMan 5.4 was used to calculate standardized mean differences and confidence intervals of 95% (CIs).

**Results:** Eight studies were included in this review with a mean methodological quality score of 7.5, which is considered high quality on the PEDro scale. The included studies suggest that more robust research with protocols and well-designed comparison groups is required to better assess changes in the body composition of older individuals through WBVE. Quantitative results were calculated, with differences in weighted means, differences in standardized means, and 95% confidence intervals (CIs).

**Conclusion:** WBVE evaluated by the studies included in this review did not demonstrate improvements in body composition, and no significant effect of WBVE was found on fat mass with standardized differences (SD = −1.92; 95% CI: –4.81 to −0.98; *p* = 0.19), lean mass with standardized mean differences (SMD = 0.06 CI 95% [–0.21; −0.33]; *p* = 0.67), or skeletal muscle mass with standardized differences (SD = 0.10; CI 95% [–1.62; 1.83]; *p* = 0.91). Therefore, to date, there is lack of adequate evidence to state that WBVE can benefit the body composition of men and women over 60 years of age. However, further studies are required to better understand the physiological impacts of WBVE on body composition.

**Systematic Review Registration:** https://www.crd.york.ac.uk/prospero/#myprosperoCRD42021248871, identifier CRD42021248871.

## 1 Introduction

According to the World Health Organization, the increase in the number of older people in the world population will accelerate in the coming decades. In 2019, the number of people aged 60 and over was 1 billion, and it is estimated that this number will increase to 1.4 billion by 2030 (World Health Organization, 2022). However, this increase in longevity does not mean years lived in good health, as the incidence rates of chronic diseases are also increasing, albeit with treatments that allow individuals to live longer with such illnesses (de Azeredo Passos et al., 2020). The aging process is associated with changes in body composition, including increased fat mass and decreased lean mass (commonly referred to as sarcopenia) (Ribeiro and Kehayias, 2014). Sarcopenia is the progressive, natural loss of skeletal muscle mass and strength as people age, resulting in impaired mobility, increased risk of morbidity, and reduced quality of life in older individuals (Ponti et al., 2020). In addition, this tendency among older people to lose skeletal muscle mass and to gain fat mass may contribute to being overweight or obese (Schoufour et al., 2021). There is evidence that obesity in the older population increases the risk of cardiovascular disease, diabetes, hypertension, and possible dementia (Fruh, 2017). A decrease in the level of physical activity may be a cause of obesity in the elderly, resulting in a decrease in energy expenditure associated with the aging process (Suryadinata et al., 2020). Changes in body composition can also affect the functionality, autonomy, and health of older individuals, so physiotherapy and physical exercise programs are recommended to contribute to improving body composition, preventing the development of chronic diseases, and maintaining functional fitness (Hall and Kahan, 2018).

Whole-body vibration exercise (WBVE) is generated when the individual performs systemic vibratory therapy (Sa-Caputo et al., 2022), in which the individual is exposed to mechanical vibrations produced on a vibrating platform (VP) in operation (van Heuvelen et al., 2021). The VP provides mechanical vibrations to the whole body through two different systems: i) vertical VP, where the base of the VP performs uniform up and down movements and ii) alternating lateral VP, where the base of the VP performs alternating lateral displacements, similar to a seesaw (Rauch et al., 2010). WBVE intensity is controlled by adjusting peak-to-peak displacement (PPD) (mm), frequency (Hz), peak acceleration (m/s^2^ or xg), and exposure time to mechanical vibration (Perchthaler et al., 2015). The physiological mechanisms involved in WBVE are still not completely understood, although it has been suggested that the potential physiological effects of WBVE on different organs/tissues could be related to possible neuromuscular responses and the tonic vibration reflex, with studies suggesting that reflex muscle contractions, adaptations, or mechanisms of postural control probably increase muscle contractions (Rittweger, 2010). Research suggests that WBVE training may positively influence neuromuscular function, contributing to increased muscle strength and physical performance (Sitjà-Rabert et al., 2012; Wang et al., 2015; Yang et al., 2021). According to these findings, Sen et al. (2020) showed improvement in functional mobility, with a decrease in the time to perform the Timed Up and Go test (TUG) after the WBVE protocol in postmenopausal women (Sen et al., 2020). WBVE may also improve body composition, as studies have shown a decrease in body fat percentage in obese postmenopausal women after a WBVE protocol (Sanchez-Gonzalez et al., 2017), and improvements in skeletal muscle mass index and physical fitness in sarcopenic older people (Chang et al., 2018), in addition to a decrease in waist circumference in subjects with metabolic syndrome after WBVE (Reis-Silva et al., 2022).

Thus, the aim of this study is to present a systematic review and meta-analysis that analyzes the effects of WBVE on body composition in people over 60 years of age.

## 2 Methods

### 2.1 Information search

This systematic review and meta-analysis followed the Preferred Reporting Items for Systematic Reviews and Meta-Analyses (PRISMA) guidelines (Page et al., 2022) and was registered on the International Prospective Register of Systematic Reviews (PROSPERO) under registration number CRD42021248871.

### 2.2 Search strategy

The searches were performed from the beginning of 2021 until February 2023 using the following strings: (“whole body vibration” OR “vibrating platform” OR “vibratory intervention” OR “vibratory therapy”) AND (“elderly” OR “older”) AND (“body composition”). Regarding the PICOS strategy, the keywords used in the search were Older (Participant) performing whole-body vibration exercise (Intervention); doing (Comparison) with other exercise modalities, placebo/sham/conventional treatments; and O (Outcome) for all reported outcomes with changes in body composition (Brown, 2020). The databases were PubMed, Scopus, Web of Science, and Embase. Secondary searches were performed on the reference lists along with citation tracking of included studies to identify other possible relevant studies.

### 2.3 Eligibility criteria

The eligibility criteria of the publications were as follows: a) randomized clinical trials; b) populations of men or women of over 60 years of age; c) participants who have undergone WBVE; d) the evaluation of the therapeutic effect of WBVE on body composition; and e) full papers in English. The exclusion criteria were as follows: a) preliminary studies; b) pilot studies; and c) conference works.

### 2.4 Study selection

During the study selection phase, all publications were exported to data management software (EndNote X9) (Gotschall, 2021), and duplicates were removed. Two independent reviewers (ARS and DBS) read the titles and abstracts of the studies and excluded works that did not meet the eligibility criteria. The remaining articles were read in full to determine their eligibility for inclusion in the present systematic review. In the case of disagreement between the reviewers, a third reviewer (ACCO) was contacted to resolve the issue. During the initial selection of the evaluated studies to be included in this review, the data were recorded on an Excel spreadsheet, and Cohen’s kappa index was used to calculate the level of agreement for aspects of the inclusion/exclusion decision (Oliveira et al., 2006).

### 2.5 Data items

Body composition is the proportion between different body components and total body mass; it can be understood as a set of components such as total body water, protein, minerals, bones, skeletal muscles, and fat, as well as an estimate of lean mass (fat-free) and fat mass (Duren et al., 2008a). Monitoring body composition can be very useful for monitoring nutritional and exercise interventions to assess changes in fat-free mass with weight gain and loss during the aging process (Kuriyan, 2018). Both aging and a sedentary lifestyle promote changes in body composition components, which can lead to cardiometabolic disorders (Chung et al., 2013). Thus, increased body fat mass is associated with cardiometabolic disorders through the development of obesity, inflammation, increased waist circumference, elevated triglyceride level, high low-density lipoprotein cholesterol, low high-density lipoprotein cholesterol, hypertension, resistance to insulin, atherosclerotic disease, and diabetes mellitus (Cruz-Jentoft and Sayer, 2019a). From another perspective, decreases in skeletal muscle mass and muscle strength have been associated with a worse prognosis for cardiometabolic health due to the development of sarcopenia, loss of functional capacity, frailty, functional impairment, falls, fractures, and even premature death (Cruz-Jentoft and Sayer, 2019b). Body composition was categorized as a primary outcome. The primary endpoint in the studies was measured as a change in body composition and was assessed using imaging techniques: i) computed tomography (CT); ii) dual-energy X-ray absorptiometry (DEXA); and iii) bioelectrical impedance analysis. In this review, the following body composition parameters were included: muscle mass of the upper leg (%); muscle cross-sectional area (%); abdominal fat mass (kg); lean body mass (kg); total fat mass (kg); trunk fat mass (kg); upper limb fat mass (kg); lower limb fat mass (kg); trunk lean mass (%); right leg lean mass (%); and whole-body skeletal muscle mass (kg).

### 2.6 Quality and risk of bias assessment

The Physiotherapy Evidence Database (PEDro) scale (Paci et al., 2022) was used to assess the methodological quality of the studies included. The PEDRo scale evaluates 11 items, and selected articles with a score equal to or greater than 7 were considered to be of ‘high’ methodological quality, those with a score of 5 to 6 as ‘regular’ methodological quality, and those with a score of 4 or less as ‘poor’ methodological quality. Two independent reviewers (ARS and DBS) assessed the methodological quality of the studies. In the case of disagreement, a third reviewer (ACCO) was contacted to resolve the issue, and Cohen’s kappa index was calculated to assess the agreement between the reviewers (Oliveira et al., 2006). The risk of bias assessment of the studies included in this review was performed using the Cochrane Collaboration risk of bias tool (Cumpston et al., 2019), which consists of seven domains, whereby each domain judges high risk of bias, uncertain risk of bias, and low risk of bias. Two independent reviewers (ARS and DBS) performed the risk of bias assessment. In the case of disagreement, a third reviewer (ACCO) was contacted to resolve the issue. Finally, the Grading of Recommendation, Assessment, Development, and Evaluation (GRADE) was used to evaluate the quality of the evidence (Marino et al., 2021).

### 2.7 Data extraction

Data extraction was performed by the same reviewers (ARS and DBS) who participated in article selection. In the case of disagreement between the reviewers, a third reviewer (ACCO) was contacted to resolve the issue. Tables were constructed with information on authors, year of publication, population, sample size, gender, age, body mass index, and the aim of study. Data on the procedures of the WBVE group and the control group, including number of sessions, frequency (Hz), total training time (min), total number of weeks of procedures, number of WBVE series, peak-to-peak displacement (mm), devices used to assess body composition, time between assessments, body composition parameters, model and type of the VP, and the results of the studies, were also extracted.

### 2.8 Statistical analysis

Review Manager (RevMan 5.4; Cochrane, London, United Kingdom) was used to compute the pooled results with 95% confidence intervals (CIs) to examine the effects of WBVE on body composition, and non-exercise control groups were used as the comparison condition. Statistical heterogeneity for the outcome in the included studies was assessed using *I* ^*2*^ statistics. According to the Cochrane Handbook, heterogeneity between 0% and 40% may not be significant, that of 30%–60% may represent moderate heterogeneity, and that of 50%–90% may indicate significant heterogeneity (Higgins et al., 2021). The mean difference (MD), standardized mean differences (SMDs), and 95% confidence interval (CI) were used to analyze studies with the same measures and units for independent and dependent variables. Sensitivity analysis was conducted by excluding trials with an assessed risk of bias to test the robustness of the pooled results (V Higgins et al., 2003; Ertefaie et al., 2016). Furthermore, treatment comparisons were performed using a z-test. The significance value was set at *p* < 0.05.

## 3 Results

**FIGURE 1:**
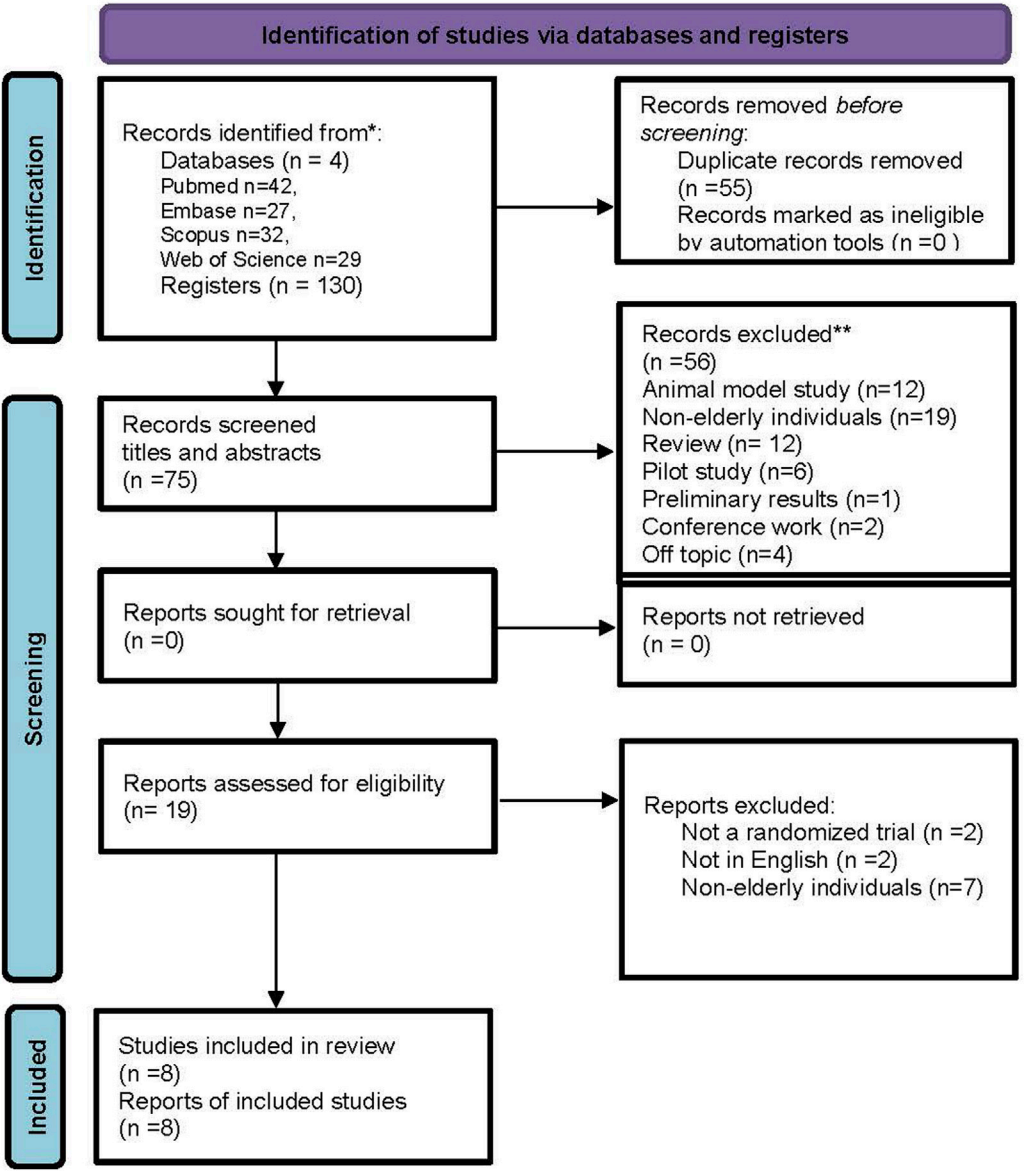
Flowchart showing the article selection process.

A total of 130 articles were initially selected, and after the exclusion of 55 duplicates, 75 abstracts were selected as relevant. During the selection process, 56 studies were excluded after reading the title and abstracts, leaving 19 studies for a full analysis. Nineteen articles were carefully read and 11 were excluded for not meeting the inclusion criteria. The reasons for exclusion were non-randomized studies, not being in English, and non-elderly population, as shown in the table in Supplementary Material S1. Finally, eight articles published from 2007 to 2021 were included in the review. The kappa index assessing the level of agreement between reviewers was equal to k = 0.92, which is considered almost perfect agreement (Oliveira et al., 2006). All are randomized clinical trials, and the selection of included studies is illustrated in the PRISMA flowchart (Page et al., 2022) (Figure 1). Five studies used only WBVE (Bogaerts et al., 2007; Machado et al., 2010; Gómez-Cabello et al., 2013; Gómez-Cabello et al., 2016; He et al., 2018), one study used 10-min warm-up and WBVE (Camacho-Cardenosa et al., 2019), and two studies used strength exercises and WBVE (Von Stengel et al., 2012; Jo et al., 2021).

### 3.1 Methodological quality and risk of bias

**FIGURE 2:**
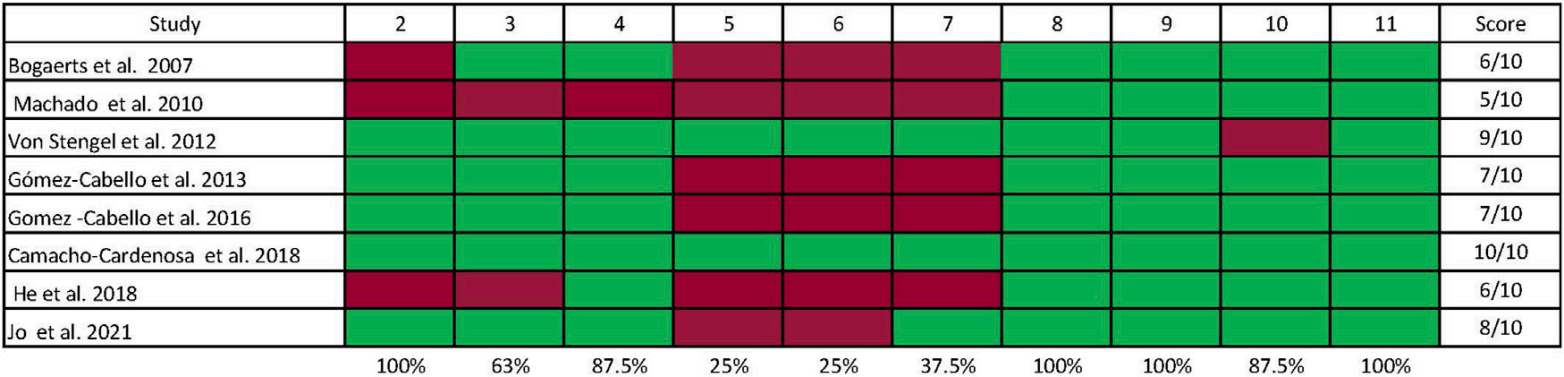
Assessment of the methodological quality of the selected studies was performed using the PEDro scale (Duren et al., 2008a), whereby the following items were judged: 1) eligibility criteria; 2) random allocation into groups; 3) allocation was hidden; 4) baseline comparability; 5) all subjects were blind; 6) there was blinding of all therapists who performed an intervention; 7) there was blinding of all evaluators; 8) adequate follow-up; 9) appropriate treatment measure or allocated control condition or “intention to treat” was performed; 10) statistical comparisons between groups are provided; 11) point assessments and measures of variability for at least one key outcome.

**FIGURE 3:**
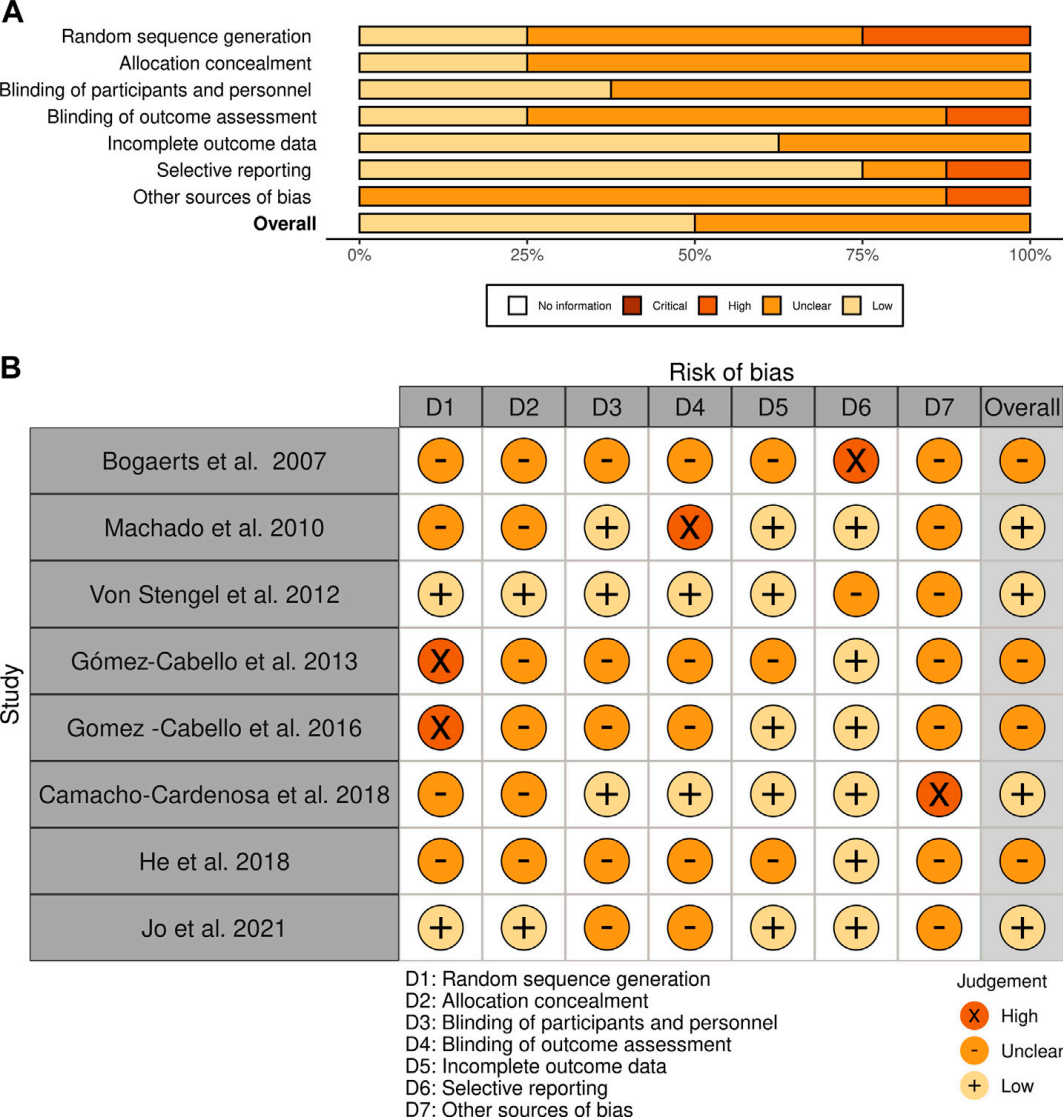
Risk of bias assessment of the studies included in this review using Cochrane. **(A)** The figure shows the evaluation of the quality of the included studies. White = no information; dark brown = critical; light brown = high; orange = unclear; pinkish = low risk of bias. **(B)** The figure presents an assessment of the quality of the included studies and the risk of bias: “+” means low risk of bias; “x” means high risk of bias; “–” means unclear risk of bias.

**FIGURE 4:**
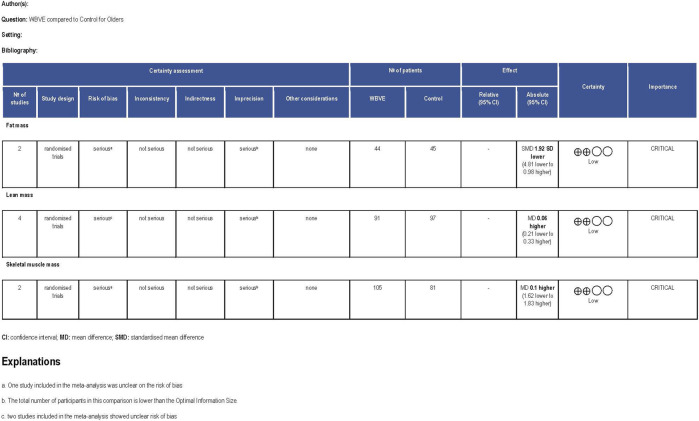
Quality of evidence assessed by GRADE.

Figure 2 presents the methodological quality assessed using the PEDro scale (Paci et al., 2022) and demonstrates that all selected studies have a high methodological quality with an average score of 7.5 (minimum of 6 points and maximum of 11). The kappa coefficient was k = 0.84, indicating an excellent agreement between reviewers. The risk of bias measured with the Cochrane Risk of Bias tool indicated that the overall bias of RCTs may be considered ‘unclear’ because, of the eight studies included in this review, four have a low risk of bias (Machado et al., 2010; He et al., 2018; Camacho-Cardenosa et al., 2019; Jo et al., 2021), and four have unclear risks (Bogaerts et al., 2007; Gómez-Cabello et al., 2013; Gómez-Cabello et al., 2016; Von Stengel et al., 2012). Detailed information on the risk of bias is presented in Figures 3A,B. GRADE showed that the quality of evidence in fat mass, lean mass, and skeletal muscle mass after a WBVE protocol in the medium and long term was considered low. The details are presented in Figure 4.

### 3.2 Participants/intervention

In total, 661 older people of both sexes and over 60 years of age participated in randomized clinical trials involving WBVE in the current review. Five studies included individuals of both sexes (Gómez-Cabello et al., 2013; Gómez-Cabello et al., 2016; He et al., 2018; Camacho-Cardenosa et al., 2019; Jo et al., 2021), whereas Machado et al. (2010) and Von Stengel et al. (2012) included only female subjects, while Marino et al. (2021) included only male subjects. In general, the number of participants varied between the studies, from 29 (Bogaerts et al., 2007) up to 200 (Gómez-Cabello et al., 2016). According to the BMI, the individuals were classified as normal body mass to obesity grade I; however, two studies did not report the BMI, namely, He et al. (2018); Machado et al. (2010). Table 1 shows the characteristics of the studies in the current review, including information on population, sample size, male or female, age, and body mass index (BMI).

**TABLE 1:** Baseline characteristics of participants.

Study	Population	Sample size	Male/female (N)	Age (year)	Body mass index (kg/m^2^) (mean ± SD)
Bogaerts et al. (2007)	Local communities	97	97	60–80	WBVE group (27.0 ± 6 0.7)
					Control group (26.9 ± 6 0.5)
Machado et al. (2010)	Community-dwelling elderly subjects	29	0/29	65–90	WBVE (28.6 ± 4.0)
					Control group (29.4 ± 4.6)
Von Stengel et al. (2012)	Independently living post-menopausal women aged 65 years and older were	151	0/151	65–76	WBVE group (26.6 ± 4.2)
					Control group (27.5 ± 5.0)
Gómez-Cabello et al. (2013)	Community-dwelling elderly subjects	49	20/29	Over 65	WBVE group (26.61 ± 3.24)
					Control group (27.71 ± 3.86)
Gómez-Cabello et al. (2016)	Community-dwelling elderly subjects	49	20/29	People over 65 years	WBVE group (27.71 ± 3.86)
					Control group (26.61 ± 3.24)
He et al. (2018)	Community-dwelling elderly subjects	200	104/96	60–83	Uninformed
Camacho-Cardenosa et al. (2019)	Senior universities and local pensioners associations	46	13/33	65	NWBVE (29.5 ± 4.8)
					HWBVE (28.9 ± 4.2)
					Control (28.9 ± 3.5)
Jo et al. (2021)	Community-dwelling elderly subjects	40	18/22	Over 65	Uninformed

### 3.3 Protocols used in the interventions

In the studies, the following squat exercises (at different degrees of knee flexion) (Bogaerts et al., 2007; Machado et al., 2010; Gómez-Cabello et al., 2016; He et al., 2018; Camacho-Cardenosa et al., 2019) were performed on the VP: i) static squat (Gómez-Cabello et al., 2013; Gómez-Cabello et al., 2016; Camacho-Cardenosa et al., 2019); ii) static and dynamic squat (Gómez-Cabello et al., 2013; Von Stengel et al., 2012); or iii) dynamic squat (He et al., 2018; Jo et al., 2021). The authors also included exercises such as calf-raises (Bogaerts et al., 2007; Machado et al., 2010; He et al., 2018; Von Stengel et al., 2012), leg abduction (Von Stengel et al., 2012), one-legged deep squats (Bogaerts et al., 2007; He et al., 2018; Von Stengel et al., 2012), and upright standing position (Bogaerts et al., 2007; Jo et al., 2021) on the VP. Three studies evaluated the effect of a long-term intervention: Bogaerts et al. (2007) (47 weeks), He et al. (2018) (47 weeks), and Von Stengel et al. (2012) (72 weeks). The vibration frequency in the studies ranged from 10 (Jo et al., 2021) to 40 Hz (Bogaerts et al., 2007; Machado et al., 2010; Gómez-Cabello et al., 2013; Gómez-Cabello et al., 2016; He et al., 2018), with a peak-to-peak displacement of 1.7 (Camacho-Cardenosa et al., 2019) to −5 mm (Bogaerts et al., 2007; Gómez-Cabello et al., 2016; Von Stengel et al., 2012). Each set of WBVE sessions lasted from 15 to 60 s (Bogaerts et al., 2007; He et al., 2018); 30–60 s (Machado et al., 2010; Camacho-Cardenosa et al., 2019); 45–60 s (Gómez-Cabello et al., 2013; Gómez-Cabello et al., 2016); and 60 s (Von Stengel et al., 2012), with a relationship observed between exposure time to mechanical vibration and rest period, except for the study by Jo et al. (2021), which seems to have used a continuous mechanical vibration exposure of 20 min. Vertical (Bogaerts et al., 2007; Machado et al., 2010; Gómez-Cabello et al., 2013; Gómez-Cabello et al., 2016; He et al., 2018; Von Stengel et al., 2012; Jo et al., 2021) and side alternating (Camacho-Cardenosa et al., 2019) vibrating platforms were used in the studies. These characteristics of the intervention and the WBVE protocols used in the studies are described in Supplementary Material S2. Table 2 shows further details on the included studies, such as the aim of the study, number of weeks, activity performed by the control group, equipment used to assess body composition, assessment time, parameters of body composition, and study results.

**TABLE 2:** Characteristics of the WBVE protocols and results on body composition parameters.

Study	Aim	Weeks	Control group	Evaluation device	Assessment time	Body composition parameter	Results
Bogaerts et al. (2007)	Measure the changes muscle mass in men between 60 and 80 years after 1 year of WBVE training	47	CG was repeatedly advised not to change lifestyle or physical activity during the project	CT	Evaluated at baseline and after 12 months.	Muscle mass of the upper leg	WBV training is as efficient as a fitness program to increase muscle mass of the upper leg
Machado et al. (2010)	Measure the changes in muscle mass tissue with 10 weeks of WBVE in older women	10	were requested not to change their lifestyle during the study or to engage in any new type of physical activity	CT	Baseline data (pretest) were collected during two testing sessions separated by 4 days; similar testing sessions were repeated 10 weeks after (post-test) the training/control period	Muscle cross-sectional area	Thigh muscle cross-sectional area increased significantly after training in VM (8.7%) and BF (15.5%)
Von Stengel et al. (2012)	Verify if vibration stimulus enhances the effect on neuromuscular performance and on body composition	72	performed a light physical exercise and relaxation program once a week in blocks of 10 weeks with breaks of 10 weeks between the blocks. Low-intensity, low-volume	DXA	Baseline (pre) and after 18 months	Body fat (%)	In the TG lean body mass, total body fat, and abdominal fat were favorably affected, but no additive effects were generated by the vibration stimulus
						Abdominal fat mass (kg)	
						Lean body mass (kg)	
Gómez-Cabello et al. (2013)	Clarify whether a short-term WBVE training has an effect on Lean mass in elderly men and women	11	not participate in any training and were asked not to change the lifestyle during the project	DXA	Baseline (pre-) before the 11 weeks of intervention and reassessed after the last session	Lean mass (Kg)	A short-term WBVE therapy is not enough to cause significant changes on LM
Gómez-Cabello et al. (2016)	Tests WBVE intervention has any effect on total and regional FM in men and women over the age of 65	11	did not participant in any training	DXA	Basiline and after 11 weeks of intervention	FM total (kg)	WBVE therapy was not an effective method for reduced fat mass in older adults
						FM trunk (kg)	
						FM upper limbs (kg)	
						FM lower limbs (kg)	
Camacho-Cardenosa et al. (2018)	Assess the predictive power of data-driven genetic predisposition scores on baseline muscular phenotypes and muscle adaptations to exercise in a healthy elderly population	11	instructed to continue with their normal daily activities for the duration of the study	DXA	At baseline (pre-) previous to the 18 weeks of intervention and reassessed 7 days after the last session	Whole body lean mass (%)	There were no significant differences between groups on body composition parameters
						Trunk lean mass (%)	
						Right leg lean mass (%)	
He et al. (2018)	Identify if WBVE training combined with exposure to norm baric cyclic hypoxia could cause higher increases in the strength parameters and muscle mass of elderly people	47	The CG was repeatedly advised not to change lifestyle or physical activity during the project	BIA	Evaluated at baseline and after 12 months	Whole-body skeletal muscle mass (kg)	showed a significant increase in skeletal muscle mass (kg) in both WBVE and control groups
Jo et al. (2021)	Verify the efficacy and safety of WBVE in improving muscle strength and physical performance before resistance exercise in older adults	4	Performed stretching (20 min), followed by the strength exercises (20 min) after a 10-min break	BIA	At baseline, post treatment and 4-week follow-up evaluation	Skeletal muscle mass (kg)	showed a significant decrease in skeletal muscle mass (kg) only in the control group
						body fat mass (kg)	

WBVE, whole-body vibration exercises; kg, kilogram; s, seconds; VP, vibrating platform; %, percentage; CT, computed tomography; BIA, bioelectrical impedance analysis; VM, vastus medialis; BF, biceps femoris; LM, lean mass; FM, fat mass.

### 3.4 Lifestyle control (physical activity and food habits) in the studies

Lifestyle control was considered in most of the studies. Bogaerts et al. (2007), Machado et al. (2010), and He et al. (2018) excluded those who participated in moderate-level physical exercise. In addition to excluding participants involved in physical exercise in the last 6 months, Camacho-Cardenosa et al. (2019) instructed participants not to get involved in exercise programs during the intervention period. Gómez-Cabello et al. (2013) and Gómez-Cabello et al. (2016) instructed participants in both groups to not change their lifestyle during the study period (eating habits or physical activity). In addition to advising the participants to not change their lifestyle, Von Stengel et al. (2012) used individual questionnaires to assess dietary intake and level of physical activity during the study period. However, Jo et al. (2021) did not report whether lifestyle control or guidance was provided to participants. In addition to lifestyle control during the intervention, another factor that could interfere with the results of the studies would be the use of other types of exercise, therapies, or diet associated with the WBVE protocol. Thus, three authors (Camacho-Cardenosa et al., 2019; Von Stengel et al., 2012; Von Stengel et al., 2012; Jo et al., 2021) associated conventional exercises with WBVE; Camacho-Cardenosa et al. (2019) used a 10-min warm-up period (5 min cycling + 5 min stretching) in the WBVE group; Jo et al. associated 20 min of strength exercises without additional load with the WBVE protocol; and Von Stengel et al. (2012) associated 20 min of dancing + 5 min of coordination + 20 min of strength training for the trunk and upper limbs with the WBVE. In addition, Camacho-Cardenosa et al. (2019) associated a situation of hypoxia during the WBVE protocol.

### 3.5 Methods used in the assessment of body composition and parameters evaluated in this systematic review

Body composition was evaluated in most studies using DEXA (Gómez-Cabello et al., 2013; Gómez-Cabello et al., 2016; Camacho-Cardenosa et al., 2019; Von Stengel et al., 2012); however, Machado et al. (2010) and Bogaerts et al. (2007) used computed tomography (CT), while Jo et al. (2021) and He et al. (2018) used BIA. These methods used to assess body composition in the studies differ in measurement principles and the assumptions required for calculations and prediction equations. Thus, there may be methodological problems or inconsistencies in estimating body composition (Duren et al., 2008b). The DEXA device is considered the most objective and accurate, the gold standard, and one of the reference methods for measuring body composition. However, the use of the DEXA system is expensive and restricted to clinical settings (Morgan and Ginnie, 2017). CT has been used to assess body composition and is considered a precise method; however, it emits radiation and is expensive (Paris, 2020). The BIA device allows measurements in a non-invasive way, with simplicity, speed, good portability, safety, and low cost; however, it requires standardized conditions for a correct evaluation and may present precision limitations, for example, in individuals with high body fat mass (Tornero-Aguilera et al., 2022). Several body composition outcomes were evaluated: i) lean body mass (kg); ii) trunk lean mass (%); iii) right leg lean mass (%); iv) cross-sectional area of skeletal muscles (*vastus medialis*, *vastus lateralis*, and *biceps femoris* muscles of the dominant leg) (%); v) body fat mass (kg); vi) abdominal fat mass (kg); vii) upper limb fat mass (kg); viii) lower limb fat mass (kg); ix) trunk fat mass (kg); x) skeletal muscle mass of the upper leg (kg); and xi) whole-body skeletal muscle mass (kg).

### 3.6 Effect of WBVE on body composition parameters

#### 3.6.1 Lean mass

Four studies evaluated the association between exposure to WBVE and lean body mass (Gómez-Cabello et al., 2013; Gómez-Cabello et al., 2016; Camacho-Cardenosa et al., 2019; Von Stengel et al., 2012), all of which used the DEXA device, the gold standard for assessing body composition. None of these studies found a significant effect or additive effect of the WBVE stimulus on lean body mass. i) Camacho-Cardenosa et al. (2019) evaluated three groups 1) the hypoxic WBVE group, who underwent vibration WBVE treatment under normobaric hypoxic conditions; 2) the normoxic WBVE group, who underwent WBVE treatment under normoxic conditions; and 3) the control group, who were instructed to continue with their normal daily activities for the duration of the study. Studies suggest that the intramuscular environment via hypoxia could be used as a method to enhance the physiological experience of resistance training as a consequence of the recruitment of motor units (Scott et al., 2014). However, there was no significant difference between the groups, even when considering WBVE under hypoxic conditions for any of the analyzed variables: (whole-body lean mass (%), trunk lean mass (%), and right leg lean mass (%)). ii) Gómez-Cabello et al. (2013) evaluated two groups: a control group (not participating in any training) and a WBVE group (squat exercise). 1) The control and WBVE groups presented similar lean mass values in all areas. No significant variations were detected for total lean mass (kg) and lean mass of arms and legs (kg). iii) Gómez-Cabello et al. (2016) also evaluated the two groups: 1) the control group (did not participate in any training) and 2) the WBVE group (static squat position) and did not detect a significant change between the groups for lean mass (kg). The only study that showed a significant change in lean mass (kg) was that of iv) Von Stengel et al. (2012) who analyzed three groups: 1) control group (relaxation exercises); 2) training group (dance/coordination/strength training); 3) WBVE (dance/coordination/strength training + WBVE). However, a significant increase was only demonstrated in the group that performed physical training without WBVE, and no significant differences were found between the training group, the WBVE + training group, and the control group.

#### 3.6.2 Skeletal muscle mass

In the present review, four studies evaluated the effects of WBVE on skeletal muscle mass: i) Machado et al. (2010) evaluated the muscle cross-sectional area (CSA) (%) of the *vastus medialis* (VM), *vastus lateralis* (VL), and *biceps femoris* (BF) muscles of the dominant leg using CT after 10 weeks of intervention. The authors evaluated two groups: 1) a control group (asked not to change their lifestyle during the study) and 2) a WBVE group (static and dynamic exercise program for lower limbs with no load on the VP). A significant increase was found for the CSA (%) of the VM and BF for the WBVE group when compared to the control group. Considering that the control group did not perform exercises, it is a little confusing to clarify whether the significant effect observed in the study was due to the physical exercises performed, the vibration (WBVE), or an association between the two (exercise + WBVE). ii) Bogaerts et al. (2007) evaluated the axial cuts of the upper part of the leg after 47 weeks of intervention in three groups: 1) WBVE group (exercise for lower limbs on VP/40 min); 2) fitness group (performed cardiovascular, resistance, balance, and flexibility exercises for approximately 1.5 h); and 3) control group (advised not to change lifestyle or physical activity during the study period). The authors observed a significant change with an increase in skeletal muscle mass (%), and these changes were different for the three groups: with a significant increase in the WBVE (3.4%, *p* .001) and FIT (3.8%, *p* .001) groups but no change in the control group. The training effect was similar in the WBVE and fitness groups, with both groups differing significantly from the control group. iii) Jo et al. (2021) evaluated skeletal muscle mass (kg) using BIA in two groups: 1) control group (stretching exercises +20 min strength exercises) and 2) WBVE group (20 min of WBVE squat +20 min strength exercises), showing no significant increase in skeletal muscle mass after 4 weeks of intervention for either of the evaluated groups. However, a significant decrease in skeletal muscle mass was observed only in the control group after 4 weeks of intervention, perhaps suggesting that WBVE prevented the loss of muscle mass. iv) He et al. (2018) evaluated skeletal muscle mass (kg) after 47 weeks of intervention using BIA in three groups: 1) fitness (aerobic, resistance, balance, and flexibility training); 2) WBVE group (squat, deep squat, wide stance squat, toe-stand, toe-stand deep, one-legged squat, and lunge on the VP); and 3) control group (did not undergo any training program). All evaluated groups showed a significant increase in muscle mass after the intervention, which may be due to the duration of the study and the fact that it was conducted with healthy older people. Although the individuals in the control group were instructed to maintain their original lifestyle during the study and to not practice any new physical activity, this condition may have been uncertain for the control group.

#### 3.6.3 Fat mass

Body fat mass was analyzed in three articles: i) Von Stengel et al. (2012) evaluated total body fat (%) and abdominal fat mass (kg) after 18 weeks of intervention in three groups: 1) control group (CG) (relaxation exercises); 2) training group (TG) (dance/coordination/strength training); and 3) training group + WBVE (VTG) (dance/coordination/strength + WBVE). The authors reported that both the training and training + WBVE groups lost body fat at a level that was statistically significant, whereas no change occurred in the control group. However, only the difference between TG and CG was significant. Likewise, there was fat loss in the abdominal region in both VTG and TG compared to CG. ii) Gómez-Cabello et al. (2016) evaluated trunk fat mass (kg), upper limb fat mass, and lower limb fat mass using DEXA after 11 weeks of intervention in two groups: 1) control group (did not participate in any training) and 2) WBVE group (static squat position on the VP). The authors showed no significant difference in total, trunk, or arm fat mass between the groups; however, there was a significant decrease in leg fat mass in both groups. iii) Jo et al. (2021) evaluated fat mass (kg) through BIA after 4 weeks of intervention in two groups: 1) control group (stretching exercises + 20 min of strength exercises) and 2) WBVE group (20 min WBVE + 20 min of strength exercises). The authors stated that neither group showed a significant result in body fat mass. None of the studies included in this review showed a positive or significant additive effect of WBVE on fat mass. Therefore, initial findings indicate that the use of the WBVE devices available on the market with the aim of weight loss or fat mass reduction should be avoided in this population.

## 4 Meta-analysis results

**FIGURE 5:**
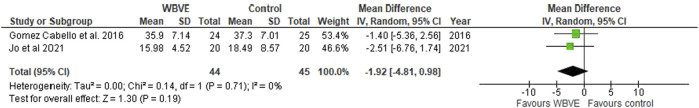
WBVE effects *versus* those of the control group on fat mass.

**FIGURE 6:**
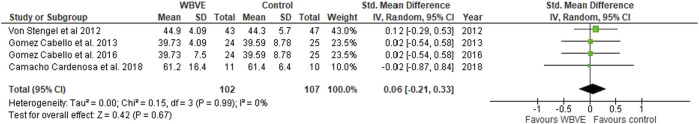
WBVE effects *versus* those of the control group on lean mass.

**FIGURE 7:**
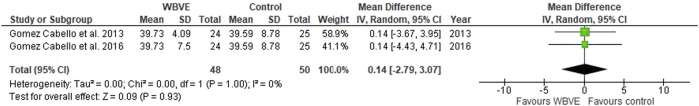
WBVE effects *versus* those of the control group on lean mass (4 weeks).

**FIGURE 8:**
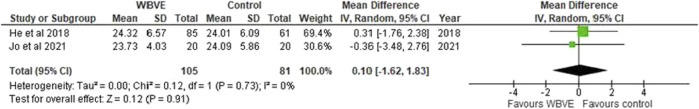
WBVE effects *versus* those of the control group on skeletal muscle mass.

The meta-analysis was performed for three body composition variables: i) fat mass (kg) in two studies, ii) lean mass (kg) in four studies and (kg) in one study; iii) whole-body skeletal muscle mass (kg) in two studies. The random-effects model meta-analysis of WBVE on fat mass in older individuals is illustrated in Figure 5. Two studies estimated the performance of a WBVE protocol on fat mass showing no significant effect (*p* = 0.19). The SD was (−1.92; 95% CI –4.81, 0.98). No significant heterogeneity was found (*I* ^2^ = 0%, *p* = 0.71). Four studies investigated the effect of WBVE on lean mass in older individuals and showed no significant difference (SMD = 0.06 95% CI [-0.21, 0.33]; *p* = 0.67). No significant heterogeneity was found (*I* ^2^ = 0%, *p* = 0.99) (Figure 6). Of these studies included in the analysis of lean mass, 2 studies presented the same intervention period (4 weeks) and were analyzed separately. The sensitivity analysis of this subgroup was in line with the baseline analysis and showed no significant change in effect size or heterogeneity, indicating agreement with the previous result (MD = 0.14 95% CI [-2.79, 3.07]; *p* = 0.93) (Figure 7). Considering the effects of WBVE on skeletal muscle mass, the meta-analysis found no significant difference in skeletal muscle mass change, using a random-effects model, MD = 0.10[–1.62, 1.83]; *p* = 0.91; and there is no statistical heterogeneity (*I* ^2^ = 0, *p* = 0.73) (Figure 8).

## 5 Discussion

The aim of this systematic review and meta-analysis was to verify the effects of conservative treatment through WBVE intervention on body composition in older individuals. The included studies provide possible evidence that WBVE can improve body composition in individuals over 60 years of age. In general, the studies used different devices to assess body composition, including more sophisticated and sensitive methods such as DEXA (considered the gold standard) or CT; however, these are often not feasible for field studies due to their high cost and the availability of other less sensitive techniques such as BIA (da Costa et al., 2022). WBVE can be considered affordable because it is available in gyms, clinics, offices, and hospitals (Gómez-Bruton et al., 2017a; Perez-Gomez et al., 2020). The portable vibrating platform model can also be easily used at home (Faes et al., 2023). In addition, the number of publications on PubMed and other databases has been increasing in recent years, demonstrating an increase in the scientific community’s interest in the applicability of WBVE under different clinical conditions (Sañudo et al., 2020; Liu et al., 2023). In general, publications on older individuals have shown benefits in muscle strength (Trans et al., 2009), flexibility (de Oliveira et al., 2022), gait (Nawrat-Szołtysik et al., 2022), physical performance (Helga Cecília Muniz et al., 2022), and body composition (Pérez-Gómez et al., 2020).

Regarding the effect of WBVE on lean mass assessed using DEXA (Table 2), Camacho-Cardenosa et al. (2019) (12 Hz/PPD 4 mm), Gómez-Cabello et al. (2013) (40 Hz/PPD 2 mm), Gómez-Cabello et al. (2016) (40Hz/PPD 4 mm), and Von Stengel et al. (2012) (25–35 Hz/PPD 4 mm) found no significant difference after a WBVE protocol. Corroborating the results of this review (Gómez-Cabello et al., 2013; Gómez-Cabello et al., 2016; Camacho-Cardenosa et al., 2019), both Pérez-Gómez et al. (2020) (12–14 Hz/PPD 3 mm), in a study on postmenopausal women, and Gómez-Bruton et al. (2017b) (30–48 Hz/PPD 2–4 mm), in a study on young swimmers, also failed to find a significant difference in lean mass. Although the protocols used were different between these studies, none showed a significant effect on lean body mass. However, Von Stengel et al. (2012) (Table 2) evaluated three training groups (training group, training group + WBVE, and control group) and demonstrated a significant increase in lean mass only for the group that underwent physical training without WBVE. The author himself reports that he had difficulty comparing his results with those of other studies because he did not use an isolated WBVE group, and he raised the hypothesis that WBVE may not promote additional gain to a conventional training group. As aging is related to significant changes in body composition, such as the reduction of lean mass, which is a risk factor for osteoporosis, functional impairment, and increased risk of falls and fractures in older individuals, in addition to increased mortality, there is significant interest in defining therapeutic possibilities to prevent or treat the loss of lean body mass (Kalyani et al., 2014). Therefore, it seems that further studies are needed to explore the ideal WBVE protocol that can increase lean body mass, possibly using different parameters to those already studied.

Machado et al. (2010) (Table 2) reported a significant increase in the cross-sectional area (%) (CSA) of the *vastus medialis* and *biceps femoris* muscles after the WBVE protocol (20–40 Hz/PPD 2–4 mm/10 weeks). Bogaerts et al. (2007) (Table 2) also demonstrated increased thigh muscle mass (%) after 1 year of WBVE (30–40 Hz/PPD 2.5/5.0 mm/1 year). Corroborating these findings, Rosenberger et al. (2017) demonstrated a significant difference in the CSA (%) of the *quadriceps femoris*, *lateral gastrocnemius*, *medial gastrocnemius*, and *triceps surae* muscles after a WBVE intervention (20–40 Hz/PPD 6–8 mm/6 weeks) in healthy young people. However, Jo et al. (2021) (Table 2) showed no change in whole-body skeletal muscle mass (kg) after a WBVE intervention (10 Hz/PPD 5 mm/4 weeks). This disagreement between the studies may be attributed to the analyzed variable of specific muscles of the lower limbs that directly received the vibratory stimulus in the studies by Machado et al. (2010), Bogaerts et al. (2007), and Rosenberger et al. (2017) and showed significant changes, while the study by Jo et al. (2021) evaluated overall whole-body muscle mass (kg) and reported conflicting results. Furthermore, the study by Jo et al. (2021) used a shorter training period (4 weeks) and a WBVE protocol with lower frequencies when compared to other studies. It is interesting to highlight that the control group showed a significant reduction in muscle mass in this study. Therefore, even though the WBVE group did not show improvement, it also did not show a worsening of muscle mass. This may suggest that a WBVE intervention could be useful for attenuating the age-associated decrease in skeletal muscle mass in older people.

The increased cross-sectional area of the skeletal muscle fibers is also known as hypertrophy, which is a multifaceted phenomenon based on mechanical stimulation and metabolic and endocrine processes (Franchi et al., 2018). The increase in skeletal muscle mass and skeletal muscle strength after WBVE can essentially be induced by neuromuscular activation. WBVE induces the “tonic vibration reflex” that is transmitted from the tendons by muscle spindle afferent neurons, resulting in the activation of large motor neurons and muscle fibers (Cochrane, 2011). The increase in skeletal muscle mass is of particular importance, as the declines in skeletal muscle mass and strength observed in the aging process can lead to physical disability and frailty (Cruz-Jentoft et al., 2019; Greco et al., 2019; Lopez et al., 2021). Furthermore, low skeletal muscle mass was positively correlated with the development of chronic diseases such as diabetes, heart disease, stroke, and chronic obstructive pulmonary disease (Vijayan, 2013).

Jo et al. (2021) (Table 2) reported no significant changes in whole-body fat mass (kg) in the intervention group (WBVE using a fixed frequency of 10 Hz) in older individuals. In line with the result of Jo et al. (2021), Gómez-Cabelloo et al. (2016) (Table 2) also concluded that WBVE did not modify fat mass with the (WBVE protocol using a fixed frequency of 10 Hz). However, Pérez-Gómez et al. (2020) observed a significant decrease in whole-body fat mass (kg) after a WBVE program using a progressive protocol frequency (12–24 Hz) in postmenopausal women. Sañudo et al. (2013), in agreement with Pérez-Gómez et al. (2020), also demonstrated a significant difference in body fat mass (kg) with a progressive WBVE intensity protocol (12–16 Hz) in individuals with diabetes. The difference in the results of these studies may be due to the type of the protocol used. The studies by Pérez-Gómez et al. (2020) and Sañudo et al. (2013) that used progressive frequency showed a modification in whole-body fat mass; however, the studies by Jo et al. (2021) and Gomez Cabello et al. (2016), which used a fixed frequency protocol, did not demonstrate this change. In addition, the population profile may also have interfered with the results as Pérez-Gómez et al. (2020) and Sañudo et al. (2013) analyzed a slightly younger population of diabetics. A nutritional plan combined with physical exercise has been shown to be useful for improving body composition and producing favorable changes in fat and skeletal muscle mass (Hernández-Reyes et al., 2019). Nevertheless, the literature justifies the decrease in fat mass after WBVE through studies carried out for assessing the inhibition of adipogenesis, increased energy expenditure, and improved muscle mass during WBVE (Cristi-Montero et al., 2013).

Furthermore, Von Stengel et al. (2012) (Table 2) demonstrated a significant decrease in abdominal fat mass (kg) for both the training group (dance/coordination/strength training) and the WBVE + training group (25–35 Hz/PPD 4 mm + dance/coordination/strength training) in an 18-month program with older individuals. The authors declared that the decrease in abdominal fat mass was similar between the training group and the vibration + training group and that there was no additional benefit for the group that practiced WBVE. Oh et al. (2022) demonstrated a significant change in abdominal fat mass after a WBVE protocol (30–40 Hz/2 mm + exercises for upper and lower limbs on the VP) after 12 weeks in obese participants but did not have a control group. Vissers et al. (2022) evaluated three groups (diet group; diet + exercise group; and diet + WBVE group) with a WBVE protocol of 30–40 Hz/PPD 2–4 mm in overweight and obese individuals and reported a significant decrease in abdominal fat, that was different after 3, 6, and 12 months of intervention for the three groups. However, after 12 months of intervention, only the diet + WBVE group showed a significant decrease in abdominal fat compared to baseline. Thus, although the three studies show a decrease in abdominal fat mass, only Vissers et al. (2022) demonstrated an additional effect of WBVE after 12 months of diet-associated intervention.

This finding could be relevant, as the more metabolically damaging abdominal fat, which surrounds critical body organs, is considered an increasingly serious risk factor for cardiovascular disease and type 2 diabetes (Wondmkun, 2020). According to Oh et al. (2022), one possibility regarding the effect of WBVE on fat mass is that the myogenesis induced by WBVE improves the recruitment of a large number of muscle fibers, resulting in increased basal metabolism and higher energy consumption. Another possibility is the activation of the sympathetic nervous system by WBVE, which could induce lipolysis in white adipose tissue, resulting in a reduction in body fat.

Changes in lifestyle factors, both in terms of dietary habits and physical exercise, can influence changes in body composition in general (Foster-Schubert et al., 2012). Thus, it would be important for studies involving investigations in body composition variables to control changes in lifestyle (Štefan et al., 2017). In the study by Camacho-Cardenosa et al. (2019), participants involved in physical exercise in the last 6 months were excluded, and participants were advised not to engage in physical exercise programs during the intervention period. Following this reasoning, in the study by Hibino et al. (2023) that assessed body composition after WBVE, participants were also advised to avoid any additional training during the study period. However, Bogaerts et al. (2007), Machado et al. (2010), and He et al. (2018) did not report additional guidance on lifestyle for the research subjects, although the older individuals who participated in moderate-level physical exercise were excluded.

### 5.1 Limitations

This systematic review and meta-analysis has some limitations, in that studies in languages other than English were not included, and only “body composition” was used as a string in this category. The frequency, amplitude, and time of exposure to mechanical vibration in the included studies were not sufficiently identified in all publications. The methods used to assess body composition also differed between studies, and some studies did not describe whether lifestyle was controlled. Therefore, these methodological differences may have influenced the divergent results between some of the included studies. Furthermore, studies with statistically significant results have not evaluated the minimal clinically important difference (MCID) to demonstrate whether the findings are also clinically effective for the patients. Thus, these factors may have influenced the divergent results between studies, inhibiting a definitive conclusion.

### 5.2 Strengths

The strengths of this study suggest that although there is a growing scientific interest in exploring this exercise modality for older individuals, a protocol that can bring significant benefits to the body composition of these individuals has not yet been precisely established.

### 5.3 Perspective and facts

The facts are that the WBVE intervention could have positive effects on the body composition of older individuals. The perspectives are that future studies, preferably randomized with blind allocation and with a higher number of participants, are required to investigate the effects of WBVE, including in older patients with other diseases, such as cardiopulmonary, cardiovascular, and neurological diseases. It would also be interesting to be able to follow the participants over the long term and compare different frequencies, peak-to-peak displacements, and types of the vibrating platform to determine the most effective protocol for these patients. 357.

## 6 Conclusion

The current systematic review and meta-analysis investigated a non-pharmacological therapy that could be used to improve body composition in the elderly. Considering the possible positive changes, the main findings are still uncertain and imprecise in relation to increased skeletal muscle mass and decreased fat mass. Therefore, there is still no adequate evidence that men and women over 60 years of age can benefit from performing a WBVE protocol. Further studies are required to better understand the physiological impacts of WBVE on body composition.

## Data Availability

The original contributions presented in the study are included in the article/Supplementary Material; further inquiries can be directed to the corresponding author.
