# Supplementary material for: Evidence of whole-body vibration exercises on body composition changes in older individuals: a systematic review and meta-analysis
Source: Front Physiol. 2023 Nov 2;14:1202613. doi: 10.3389/fphys.2023.1202613 (PMC10652794; doi:10.3389/fphys.2023.1202613)
Supplement: Supplementary file 1 [file Table1.docx]

| Table 1. Baseline characteristics of participants | | | | | |
| --- | --- | --- | --- | --- | --- |
| Study | Population | Sample Size | Male/female (N) | Age (year) | Body mass  index (kg/m2) (mean± SD) |
| Bogaerts et al. 2007 | Local communities | 97 | 97 | 60 - 80 | WBVE group (27.0 ± 6 0.7)   Control group (26.9 ± 6 0.5) |
| Machado et al. 2010 | Community-dwelling elderly subjects | 29 | 0/29 | 65–90 | WBVE (28.6 ± 4.0) Control group (29.4 ± 4.6) |
| Von Stengel et al. 2012 | Independently living post-menopausal women aged 65 years  and older were | 151 | 0/151 | 65–76 | WBVE group (26.6 ± 4.2) Control group (27.5 ± 5.0) |
| Gómez-Cabello et al. 2013 | Community-dwelling elderly subjects | 49 | 20/29 | Over 65 | WBVE group (26.61 ± 3.24)  Control group (27.71 ± 3.86) |
| Gómez-Cabello et al. 2016 | Community-dwelling elderly subjects | 49 | 20/29 | People over  65 years | WBVE group (27.71 ± 3.86) Control group (26.61 ± 3.24) |
| He et al. 2018 | Community-dwelling elderly subjects | 200 | 104/96 | 60 - 83 | Uninformed |
| Camacho-Cardenosa et al. 2019 | Senior universities and local pensioners associations | 46 | 13/33 | 65 | NWBVE (29.5 ± 4.8) HWBVE (28.9 ± 4.2) Control (28.9 ± 3.5) |
| Jo et al. 2021 | Community-dwelling elderly subjects | 40 | 18/22 | Over 65 | Uninformed |
