# Supplementary material for: Evidence of whole-body vibration exercises on body composition changes in older individuals: a systematic review and meta-analysis
Source: Front Physiol. 2023 Nov 2;14:1202613. doi: 10.3389/fphys.2023.1202613 (PMC10652794; doi:10.3389/fphys.2023.1202613)
Supplement: Supplementary file 2 [file Table2.docx]

Table 2. Characteristics about the WBVE protocols and results on body composition parameters.

| Study | Aim | Weeks | Control group | Evaluation device | Assessment Time | Body composition parameter | Results |
| --- | --- | --- | --- | --- | --- | --- | --- |
| Bogaerts et al. 2007 | Measure the changes muscle mass in men between 60 and 80 years after 1 year of WBVE training | 47 | CG was repeatedly advised not to change lifestyle or physical activity during the project | CT | Evaluated at baseline  and after 12 months. | Muscle mass of the upper leg | WBV training is as efficient as a fitness program to increase muscle mass of the upper leg |
| Machado et al.  2010 | Measure the changes in muscle mass  tissue with 10 weeks of WBVE in older  women | 10 | were requested not to change their lifestyle  during the study  or to engage in any new type of physical activity | CT | Baseline data (pretest) were collected during two testing sessions separated by 4 days; similar testing sessions were repeated 10 weeks after (post-test)  the training/control period. | Muscle cross-sectional area | Thigh muscle cross-sectional area increased significantly after training in VM (8.7%) and BF (15.5%). |
| Von Stengel et al. 2012 | Verify if vibration stimulus enhances the effect on neuromuscular performance and on body composition | 72 | performed a light physical exercise and relaxation program once a week in blocks of 10 weeks with breaks of 10 weeks between the blocks. Low-intensity, low-volume. | DXA | Baseline (pre) and after 18 months. | Body fat (%)  Abdominal fat mass(kg)  Lean body mass (kg) | In the TG lean body mass, total body fat, and abdominal fat were favorably affected, but no additive effects were generated by the vibration stimulus. |
| Gómez-Cabello et al. 2013 | Clarify whether a short-term WBVE training  has an effect on Lean mass in elderly men and women | 11 | not participate in  any training and were asked not to change the lifestyle during the project | DXA | Baseline (pre-) before the 11 weeks of intervention and reassessed after the last session | Lean mass (Kg) | A short-term WBVE therapy is not enough to cause significant changes on LM |
| Gómez-Cabello et al. 2016 | Tests WBVE intervention has any effect on total and regional FM in men and women over the age of 65. | 11 | did not participant in any training. | DXA | Basiline and after 11 weeks of intervention | FM total (kg)  FM trunk (kg)   FM upper limbs (kg)   FM lower limbs (kg) | WBVE therapy was not an effective method for reduced fat mass in older adults |
| Camacho-Cardenosa et al. 2018 | Assess the predictive power of data-driven genetic predisposition scores on baseline muscular phenotypes and muscle adaptations to exercise in a healthy elderly population | 11 | instructed to continue with their normal daily activities for the duration of the study | DXA | At baseline (pre-) previous to the 18 weeks of intervention and reassessed 7 days after the last session | Whole body lean mass (%)   Trunk lean mass (%)   Right leg lean mass (%) | There were no significant differences between groups on body composition parameters. |
| He et al. 2018 | Identify if WBVE training combined with exposure to norm baric cyclic hypoxia could cause higher increases in the strength parameters and muscle mass of elderly people | 47 | The CG was repeatedly advised not to change  lifestyle or physical activity during the project | BIA | Evaluated at baseline  and after 12 months | Whole-body skeletal muscle mass (kg) | showed a significant increase in skeletal muscle mass (kg) in both WBVE and control groups |
| Jo et al. 2021 | Verify the efficacy and safety of WBVE in improving muscle strength and physical performance before resistance exercise in older adults | 4 | performed stretching (20 min), followed by the strength exercises (20 min) after a 10-min break. | BIA | At baseline, post treatment and 4-week follow-up evaluation | Skeletal muscle mass (kg)  body fat mass (kg) | showed a significant decrease in skeletal muscle mass (kg) only in the control group |

WBVE: whole body vibration exercises; kg: kilogram; s: seconds; VP: vibrating platform; %: percentage; CT: computed tomography; BIA: Bioelectrical impedance analysis; VM vastus medialis; BF biceps femuralis; LM: lean mass; FM: fat mass
